# Impact of pulse duration on cardiac electroporation: nanosecond pulses enhance cardiomyocyte selectivity and promote a Raman-detected shift towards apoptotic cell death

**DOI:** 10.1093/europace/euaf217

**Published:** 2025-09-13

**Authors:** Pamela W Sowa, Aleksandra Mariyanats, Aleksander Kiełbik, Anne-Katrin Rohlfing, Vitalij Novickij, Ferdinand Kollotzek, Manuel Sigle, Julia Marzi, Katja Schenke-Layland, Oliver Borst, Meinrad P Gawaz

**Affiliations:** Department of Cardiology and Angiology, University Hospital Tübingen, Otfried-Müller-Straße 10, Tübingen 72076, Germany; Institute of Biomedical Engineering, Department for Medical Technologies and Regenerative Medicine, Eberhard Karls University of Tübingen, Tübingen, Germany; Department of Urology, University Hospital Tübingen, Tübingen, Germany; Department of Cardiology and Angiology, University Hospital Tübingen, Otfried-Müller-Straße 10, Tübingen 72076, Germany; Institute of High Magnetic Fields, Vilnius Gediminas Technical University, Vilnius, Lithuania; Department of Immunology and Bioelectrochemistry, State Research Institute Centre for Innovative Medicine, Vilnius, Lithuania; Department of Cardiology and Angiology, University Hospital Tübingen, Otfried-Müller-Straße 10, Tübingen 72076, Germany; DFG Heisenberg Group Cardiovascular Thrombo-Inflammation and Translational Thrombocardiology, University of Tübingen, Tübingen, Germany; Department of Cardiology and Angiology, University Hospital Tübingen, Otfried-Müller-Straße 10, Tübingen 72076, Germany; Institute of Biomedical Engineering, Department for Medical Technologies and Regenerative Medicine, Eberhard Karls University of Tübingen, Tübingen, Germany; NMI Natural and Medical Sciences Institute at the University of Tübingen, Reutlingen, Germany; Institute of Biomedical Engineering, Department for Medical Technologies and Regenerative Medicine, Eberhard Karls University of Tübingen, Tübingen, Germany; NMI Natural and Medical Sciences Institute at the University of Tübingen, Reutlingen, Germany; Department of Cardiology and Angiology, University Hospital Tübingen, Otfried-Müller-Straße 10, Tübingen 72076, Germany; DFG Heisenberg Group Cardiovascular Thrombo-Inflammation and Translational Thrombocardiology, University of Tübingen, Tübingen, Germany; Department of Cardiology and Angiology, University Hospital Tübingen, Otfried-Müller-Straße 10, Tübingen 72076, Germany

**Keywords:** Nanosecond pulsed electric fields, Microsecond pulsed electric fields, Electroporation, Cardiomyocyte, Permeabilization, Raman spectroscopy

## Abstract

**Aims:**

Pulsed field ablation (PFA), a cardiac ablation technique using microsecond pulsed electric fields (µsPEF), is widely used in clinical settings, while nanosecond pulsed electric fields (nsPEF) have recently entered clinical trials. Selective ablation of cardiomyocytes over endothelial cells is critical to prevent adverse remodelling, arrhythmias, and thrombosis, yet comparative data on nsPEF vs. µsPEF remain limited. This study investigates the cytotoxic effects and cell death mechanisms induced by nsPEF and µsPEF in cardiac and endothelial cells.

**Methods and results:**

Human cardiomyocytes and endothelial cells were exposed to varying electric field intensities with nsPEF and µsPEF using custom-built automated setup to assess permeabilization and cell death. Raman spectroscopy evaluated biochemical changes in cardiomyocytes following electroporation. *Ex vivo* epicardial ablation was performed on murine hearts using customized electrodes. Maximal cardiomyocyte death occurred 24 h after both pulse types *in vitro*. *Ex vivo*, both pulse types produced visible myocardial lesions as early as 1 h post-exposure, with lesion size progressively increasing up to 4 h. Microsecond pulsed electric fields induced significantly greater endothelial damage (ED_50_: 1.18 kV/cm) than damage to cardiomyocytes (ED_50_: 1.28 kV/cm), whereas nsPEF affected both cell types equally (ED_50_: 7.27 kV/cm vs. 7.24 kV/cm). Raman spectroscopy analysis of exposed cells indicated that µsPEF predominantly triggered necrotic or unregulated cell death, while nsPEF exposure was associated with regulated, apoptotic cell death.

**Conclusion:**

Pulse duration critically determines electroporation selectivity and downstream death pathways. Nanosecond pulsed electric fields favoured regulated cell death and cardiomyocyte selectivity, highlighting its potential to improve the safety and durability of PFA.

## Graphical Abstract

**Graphical Abstract:**
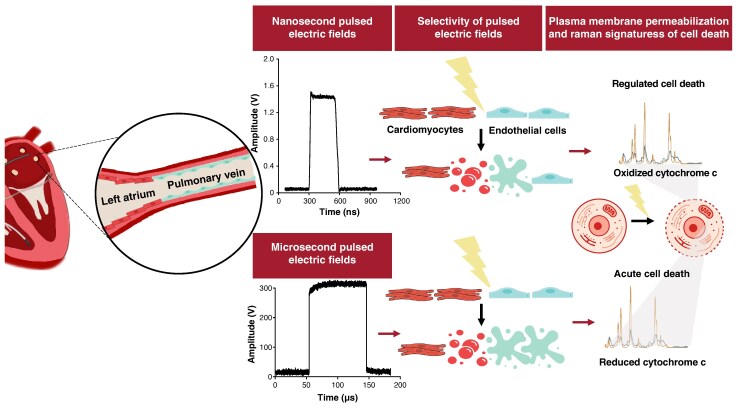

What’s new?

- This study is the first to systematically compare nanosecond pulsed electric fields (nsPEF) and microsecond pulsed electric fields (µsPEF) for cardiac ablation, filling a key knowledge gap as nsPEF enters clinical trials.
- Nanosecond pulsed electric fields promoted cardiomyocyte-selective killing with reduced endothelial damage, supporting its potential to minimize adverse remodelling and thrombosis.
- Cardiomyocytes showed delayed cell death kinetics compared to endothelial cells, while nsPEF exposure maintained a tighter coupling between membrane permeabilization and lethality—potentially reducing myocardial stunning.
- Raman spectroscopy revealed that nsPEF ablation favours oxidized cytochrome c-associated apoptotic pathways, while µsPEF shifts cytochrome c to its reduced, apoptosis-inhibiting form, indicating difference in cell death mechanisms.

## Introduction

Electroporation, also known as electropermeabilization,^1^ uses short, high-intensity electric fields to create temporary pores in cell membranes.^2^ This method has become essential in medical applications, particularly for tissue and tumour ablation. Initial efforts to use direct current for cardiac ablation faced challenges due to tissue excitation, leading to proarrhythmic activity and muscle contractions.^3^ As a result, radiofrequency ablation using alternating current (AC) emerged, relying on thermal injury for tissue ablation.^4–6^ Advances in electroporation-based tumour therapies refined pulse parameters, reducing tissue excitation and enabling more precise ablation.^7,8^ These advances have since been adapted for cardiac ablation, with pulsed field ablation (PFA) emerging as a promising technique^9^ shown potential in clinical trials^10–17^ for offering faster procedures and reducing complications, such as oesophageal injury.^5,18^

A key advantage of pulsed electric fields (PEFs) over other ablation technologies is their ability to selectively target cellular compartment of tissue, primarily affecting cell membranes while preserving the extracellular matrix. Further understanding the differential effects of PEF on various cell types is more complex. Most of our current knowledge about cellular sensitivity to PEF comes from *in vitro* studies, particularly in cancer ablation, which consistently demonstrate variable responses among different tumour types.^19–22^

There is limited knowledge about how specific physiological and morphological characteristics influence cellular sensitivity to PEF. Earlier studies, especially those using electrical circuit models, suggested that larger cells or cells with larger nuclei may be more vulnerable to damage.^21,23,24^ However, experimental evidence has been inconsistent.^22,25^ Research by Gianulis *et al*.^22^ highlighted that the sensitivity of cells to nsPEF does not strongly correlate with factors like cell size, shape, or metabolic rate, nor with the extent of electroporative membrane damage. These findings underscore the complexity of predicting electroporation outcomes. Nevertheless, most studies agree that nanosecond pulses produce greater variability in cellular responses than microsecond pulses,^26–28^ allowing for more precise targeting of specific cell types compared to conventional methods.

The selectivity of short electric pulses for cardiomyocytes during cardiac ablation is crucial to prevent tissue remodelling and avoid excessive damage to endothelial cells, which could create new substrates for arrhythmias.^29–31^ Endothelial dysfunction after ablation contributes to oxidative stress and elevated reactive oxygen species (ROS), disrupting cardiac ion currents and Ca²⁺ regulation,^32^ which can lead to myocardial fibrosis and electrical uncoupling.^33^ Furthermore, endothelial damage can cause prothrombotic changes, such as mural thrombi and fibrosis in the atrial endocardium.^34,35^ Clinically, biomarkers of endothelial dysfunction—such as elevated ADMA levels,^36^ reduced brachial FMD,^37^ and low RH-PAT index^38^—have been linked to atrial fibrillation recurrence after cardiac ablation.

While cardiomyocytes are generally considered more sensitive to electroporation than endothelial cells, definitive data remain scarce, and the differential effects of nsPEF and µsPEF on cardiac cells are not well understood. This study aims to compare the mechanisms and cytotoxic efficiency of nsPEF and µsPEF on cardiac and endothelial cells, revealing differences in their ability to selectively permeabilize and kill cardiomyocytes while sparing endothelial cells.

## Methods

### Cell cultures

Experiments were conducted using the AC16 human cardiomyocytes (Cytion, Eppelheim, Germany) and human umbilical vein endothelial cells (HUVEC, C-12205; PromoCell, Heidelberg, Germany). Cells were cultured at 37°C in a 5% CO₂ atmosphere in their respective recommended media and expanded to 80–90% confluency. Human umbilical vein endothelial cells were cultured in EC growth medium (C-22010; PromoCell, Heidelberg, Germany) and AC16 cells in Dulbecco’s Modified Eagle Medium/Nutrient Mixture F-12 (DMEM/F12; Capricorn Scientific, Ebsdorfergrund, Germany). All media supplemented with 2 mM L-Glutamine (Thermo Fisher Scientific, Waltham, MA USA), 10% FBS (Thermo Fisher Scientific, Waltham, MA USA), and 1× penicillin-streptomycin (Thermo Fisher Scientific, New York, USA).

### Setup for electroporation of cell monolayers

Two days before PEF exposure, cells were seeded onto 24-well plates at a density of 0.15 × 10^6^ cells per well, ensuring homogeneous monolayer formation. Cells were cultured on nanofiber plates (Nanofiber Solutions 9602, Darmstadt, Germany) pre-coated with 2 µg/cm² of human fibronectin (Merck, Darmstadt, Germany). To standardize conditions, 10 min before each experiment, the specific growth medium for each cell line was replaced with DMEM containing 10% FBS, maintaining consistent cell viability and uniform conductivity during electroporation.

### Electrode arrays

We developed three custom electrode arrays for PEF delivery:

- For nanosecond pulses, two parallel stainless steel rods (0.9 mm outer diameter, 1.9 mm centre-to-centre distance).
- For microsecond pulses, stainless steel rods with 1.65 mm (cathode) and 0.5 mm (anode) outer diameters, spaced 1.85 mm apart. To minimize gas evolution during microsecond pulse trains, we enlarged the cathode, reducing current density at its surface and preventing bubble formation, which could otherwise distort the electric field. This decrease in current density helps alleviate gas evolution at the electrode, as noted in previous studies where trains of long pulses (≥100 μs) at lethal intensities resulted in significant bubble formation at the cathode.^8,39^
- For *ex vivo* studies, an additional array with tungsten rods (0.5 mm diameter, 1 mm centre-to-centre spacing) was used.

### Electroporation procedure

Electric field parameters—pulse number, frequency, and intensity—were optimized through preliminary experiments to create comparable lesions across nanosecond and microsecond pulses.

**Figure 1:**
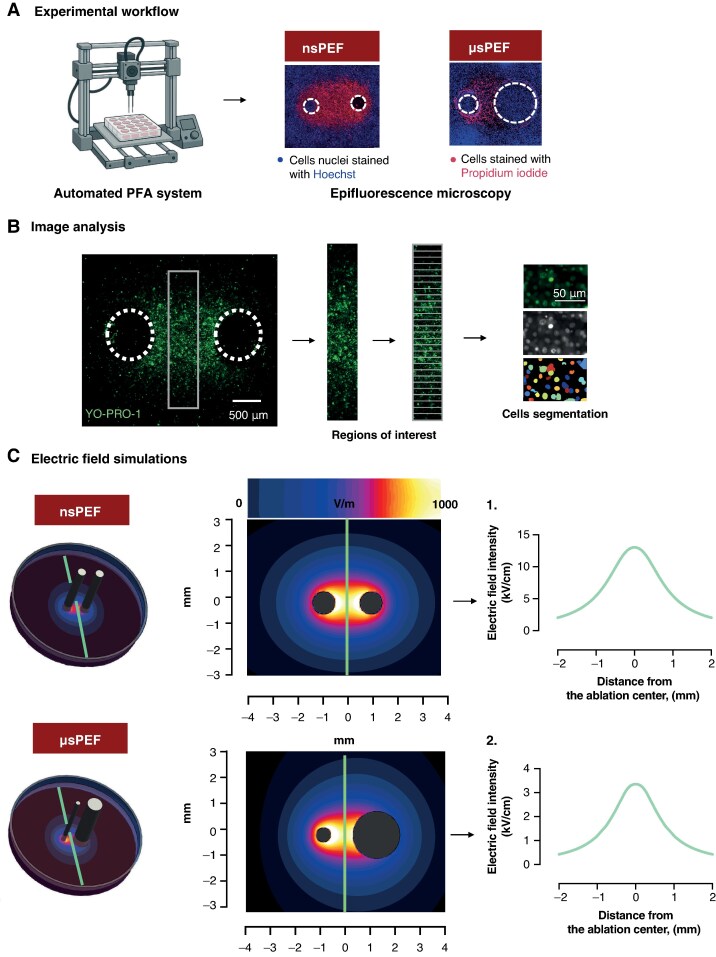
Experimental workflow, image analysis, and electric field simulation. (*A*) Cell exposure to PEFs, staining, and imaging: monolayers of cells were exposed to nsPEF or µsPEF using a high-throughput automated system. Electrodes were positioned perpendicular to the cell monolayer to ensure precise delivery of electric pulses. Pulses were applied upon contact with the cell surface, with waveform parameters, including shape, duration, frequency, and voltage, monitored via an oscilloscope to ensure accuracy. Following exposure, cells were stained with Hoechst 33342 (all nuclei), PI (dead cells), and YO-PRO-1 (permeabilized cells). These fluorescent markers enabled visualization and quantification of electric lesions via epifluorescence microscopy. (*B*) Image analysis and ROI selection: fluorescence images included white circles marking electrode contact sites. To evaluate cellular responses across a gradient of field strengths, a rectangular region between the electrodes footprints, perpendicular to the line connecting their centres, was analysed. Multiple ROIs were defined within this area. Automated image analysis software was used to identify nuclei and calculate the ratio of PI- or YO-PRO-1-positive cells to Hoechst-stained nuclei, quantifying cell death and membrane permeability. (*C*) Electric field simulation: electric field distributions were simulated using a finite element solver based on the applied voltages: 1.4 kV between symmetrical electrodes (nsPEF) and 0.3 kV between asymmetrical electrodes (µsPEF). Field intensity was estimated for each ROI within the monolayer. Graphs 1 and 2 display the electric field distribution along the green lines, respectively, each perpendicular to the axis connecting the electrode centres.

A modified Anet A8 3D printer (Shenzhen Anet Technology Co., China) with a custom electrode holder was used for precise positioning of electrodes perpendicular to the cell monolayers (*Figure 1A*).^8^ We described already the exact sequence of events during the electroporation with 3D printer in our previous studies.^40,41^

### Pulse generation and monitoring

A pulse generator, constructed at the Institute of High Magnetic Fields (VGTU, Vilnius, Lithuania), delivered pulses from 100 ns to 1 ms. For nsPEF, trains of 200 pulses at 1.4 kV were delivered at 10 Hz, while for µsPEF, 20 pulses at 300 V with 100 μs duration were delivered at 1 Hz. Pulse shape and amplitude were monitored with a TBS1052C oscilloscope (Tektronix, Beaverton, OR, USA).

### *In vitro* protocol

For fluorescence imaging, cells were incubated with 2.25 μM Hoechst-33342 (Thermo Fisher Scientific), a membrane-permeant nucleic acid dye, 1 h before exposure. To assess cell death, 75 μM propidium iodide (PI) (Thermo Fisher Scientific) solution was added at intervals from 2 to 60 h post-exposure, marking permanently permeabilized (dead) cells.^42–45^ Propidium iodide–positive cells also showed Hoechst positivity, appearing pink when channels were merged.

YO-PRO-1 (Thermo Fisher Scientific), a nucleic acid dye sensitive to membrane permeabilization, was used for nanopore detection. The dye was added right before electroporation. Fluorescence microscopy was performed using a Nikon Eclipse Ti2 microscope (Nikon Instruments Europe BV) with a 4×, 0.3 NA objective. The samples were imaged using DAPI, Cy3, and FITC filter sets for Hoechst, PI, and YO-PRO-1, respectively.

### Image analysis

For image analysis post-PEF exposure, a rectangular region of interest (ROI) (0.5 mm × 3.7 mm) was defined between electrode centres perpendicular to the line connecting the electrodes (see *Figure 1B*). Using CellProfiler software (version 4.2.5, Broad Institute, Cambridge, MA), the primary ROI was divided into smaller ROIs. Within each, the number of cells stained with PI, YO-PRO-1, and Hoechst was counted. The percentage of dead cells was calculated as the ratio of PI-positive cells to the total count. Regions of interest with minimal electric field exposure served as sham controls.

Cells were classified as YO-PRO-1 positive only when their fluorescence intensity was more than 30% above the background level within an area matching the minimum nuclear size defined by Hoechst staining (7 µm). Cells showing lower YO-PRO-1 fluorescence or a fluorescent area smaller than the nuclei were considered non-permeabilized.

### Electric field simulations

Electric field distribution was simulated using Sim4Lifelite v7.3.0 software (Zurich Med Tech, Switzerland), matching experimental electrode positions (*Figure 1C*). Electric field values were calculated at a plane 5 µm above the well bottom for 1 V applied between electrodes and then scaled to the applied voltage (1.4 kV for nsPEF, 300 V for µsPEF).

### *Ex vivo* protocol

All animal experiments were conducted in accordance with the Directive 2010/63/EU of the European Parliament on the protection of animals used for scientific purposes and were approved by the local authorities (Regierungspräsidium Tübingen) under animal protocol no. M03/19 M, following the ARRIVE guidelines. Mice were housed under a 12 h light/12 h dark cycle at temperatures of 18–23°C with 40–60% humidity. Water and food were provided *ad libitum*. To minimize the use of experimental animals, tissue samples were repurposed from experiments conducted as part of other projects.

Mice were euthanized by cervical dislocation under isoflurane anaesthesia (5% v/v), and hearts were extracted. Electrodes positioned 1 mm apart were inserted into the epicardium, spanning the ventricular wall. Ablation was performed with 300 ns, 200-pulse nsPEF at 10 Hz, and 100 µs, 20-pulse µsPEF at 1 Hz.

To assess lesion size and progression, hearts were incubated at 37°C in pre-warmed Claycomb medium (Merck, Darmstadt, Germany) for 0.5, 1, 2, or 4 h post-ablation, then immersed in 23,5-triphenyltetrazolium chloride (TTC) solution (10 mg/mL, prepared by dissolving 0.25 g TTC in 25 mL dPBS) for 20 min to stain viable tissue. Tissues were subsequently fixed in formaldehyde and imaged using a high-resolution scanner (ViewPix 700, BiobTep). This experimental workflow allowed temporal assessment of electroporation-induced lesion development. During the first 4 h post-ablation, lesions showed sharp, well-demarcated borders with clear separation between TTC-positive (viable) and TTC-negative (non-viable) tissue. We did not observe loss of TTC staining outside the ablation zone in this interval; the gradual, spatially confined enlargement of the lesion therefore likely reflects biologically meaningful, electroporation-mediated injury. Regions outside the ablation zone retained TTC staining for up to 4 h, supporting the specificity of the observed changes. Beyond 4 h, progressive pallor developed in previously unaffected regions, consistent with declining viability due to absent perfusion. Accordingly, we restricted quantitative analyses to the first 4 h to capture early injury dynamics relevant to clinical PFA while minimizing confounding artefacts.

Additionally, it should be noted that flattening of the heart tissue during scanning artificially increased its apparent dimensions, potentially leading to overestimation of lesion size.

### Image analysis hearts

Heart image analysis involved the detection and segmentation of TTC-stained lesions using Ilastik software. Initially, a training phase was conducted where representative regions in selected images were manually annotated to distinguish lesions from background tissue. These annotations were used to train Ilastik’s classifier to identify patterns based on pixel intensity and texture features. Following training, the classifier automatically segmented all images, distinguishing lesions from surrounding tissue according to the learned patterns. Ilastik assigned probability scores to each pixel, producing greyscale probability maps, which were subsequently exported and analysed in CellProfiler to quantify lesion size and shape. Morphometric parameter, the form factor, was used to quantify the uniformity and compactness of the electroporation-induced lesion in *ex vivo* heart tissue. Specifically, it is defined as form factor = 4π × area/(perimeter)^2^. This parameter was calculated from binary lesion masks generated using Ilastik-based segmentation of TTC-stained tissue sections. A form factor value close to 1.0 indicates a more circular and uniform lesion, whereas lower values suggest irregular or fragmented lesion shapes.

### Raman microspectroscopy and imaging

Forty-eight hours prior to Raman imaging, cells were seeded into removable eight-well chambers (Ibidi, Gräfelfing, Germany) pre-coated with human fibronectin, electroporated, and incubated for 4 h. After incubation, cells were fixed with 4% paraformaldehyde solution. Raman spectroscopy of fixed cells was performed using the confocal Raman microspectrometer WITec alpha 300R (WITec GmbH, Ulm, Germany) that was equipped with a green laser (532 nm) and a CCD spectrograph with a grating of 600 g/mm. The microspectrometer was managed by Control 6 software (WITec GmbH). Images of 50 × 50 µm frame size were acquired at a laser power of 50 mW, an integration time of 0.1 s per spectrum, a pixel resolution of 1 × 1 µm, and at least as triplicates. Cells were submerged in PBS during the measurement and imaged with 63× dipping objective (W Plan-Apochromat, Carl Zeiss GmbH, Jena, Germany).

### Spectral processing and multivariate data analysis

Pre-processing of obtained spectra included four steps: (i) removing artefacts originating from cosmic rays; (ii) cropping spectra within the wavenumber range 327–3045 cm^−1^; (iii) subtracting the spectral background; and (iv) normalizing of the area under every spectrum to 1.

Characteristic spectra of cellular components were identified by true component analysis (TCA)—mathematical fitting model based on a non-negative matrix factorization, implemented within the Project 6 software (WITec GmbH, Ulm, Germany). True component analysis extracts the dominant spectral components present in the dataset and visualizes their localization using false-colour intensity distribution heat maps.

Principal component analysis (PCA) (The Unscrambler X, CAMO Software AS, Oslo, Norway) of acquired data was used to identify and interpret changes in molecular fingerprints of obtained cellular components between the groups of samples ablated by nsPEF and µsPEF. Principal component analysis was performed using the NIPALS algorithm. Spectral information was visualized in 2D scattered plots, information on the contribution to the variation in the dataset was visualized and interpreted based on the loadings plots, and statistical comparison was visualized in box plots.

### Statistical analysis

Different experimental conditions (length of pulses) were randomized and tested across each cell line. For experiments evaluating the electric field strength response, a single experiment yielded 28 measurements, with one measurement taken per ROI. The data from two ROIs, symmetrically located relative to the ablation centre (e.g. the two most peripheral ROIs), were averaged to yield 14 distinct data points.

Electric field strength-response data were analysed by fitting a sigmoidal curve using the Hill equation.^46^ Curve fits, with 95% confidence intervals, were generated in GraphPad software (Version 10.1.1, Boston, MA, USA). This non-linear regression approach enabled calculation of the electric field dose that induces a 50% response in cells (ED₅₀) for each electroporation protocol. ED₅₀ values across protocols were then compared using an extra sum-of-squares F test.

The dynamics of cell death were quantified by determining the percentage of dead cells within ROIs at specified time intervals. A paired *t*-test was used to compare data from the same ROI over an extended period, allowing for accurate assessment of changes in cell death over time. The time point showing the most significant difference from the initial cell death measurement (taken 2 h post-exposure) was identified as the endpoint for cell death.

In *ex vivo* studies, lesion analysis data are presented as mean ± SEM, derived from at least four independent experiments per pulsing protocol. Comparisons between protocols were conducted using unpaired *t*-test or one-way ANOVA with Tukey’s correction for multiple comparisons. Statistical significance is indicated by asterisks: ****P < 0.0001, ***P < 0.001, **P < 0.01, and *P < 0.05.

## Results

### Cardiomyocyte showed delayed cell death after nanosecond pulsed electric fields and microsecond pulsed electric fields compared to endothelial cells

**Figure 2:**
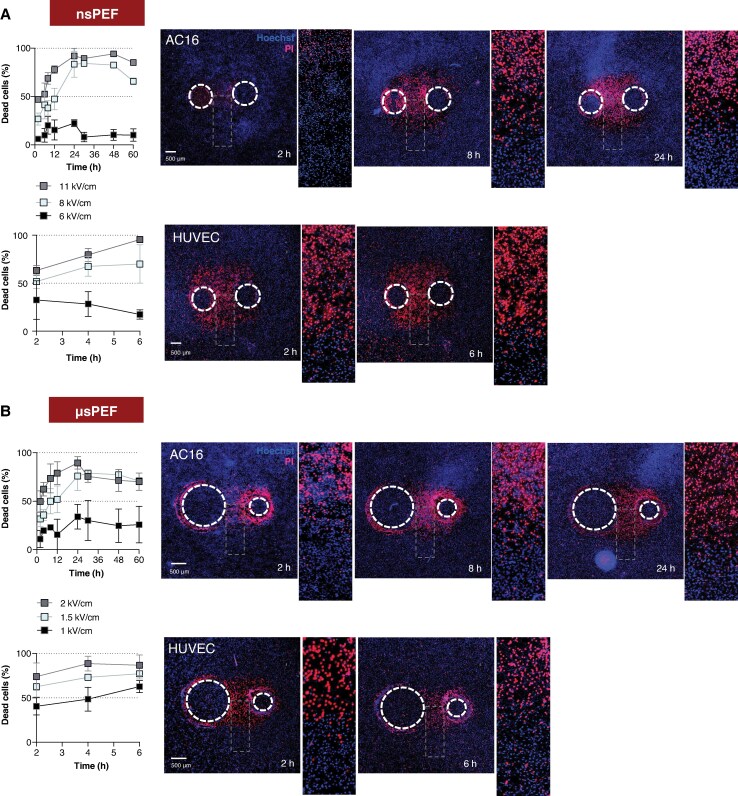
Time course of cell death in human cardiomyocytes and endothelial cells following nsPEF and µsPEF exposure. (*A* and *B*) Time-dependent cell death in human cardiomyocytes (AC16) and endothelial cells (HUVEC) exposed to nanosecond pulsed electric fields (nsPEF: 200 pulses, 10 Hz, 300 ns, 1.4 kV; *A*) or microsecond pulsed electric fields (µsPEF: 20 pulses, 1 Hz, 100 µs, 300 V; *B*). Graphs depict the percentage of dead cells within regions exposed to varying electric field strengths at different time points. Data are presented as mean ± SEM from four independent experiments per time point. Cardiomyocyte death increased significantly over the first 24 h after both nsPEF and µsPEF exposure, with an apparent decline between 24 and 60 h likely reflecting repopulation of the ablated area by viable cells and/or detachment of dead cells. Endothelial cells exhibited an earlier onset of death, with marked increases evident as early as 2 h after both treatments. Representative fluorescence microscopy images (right side of each panel) illustrate monolayer morphology following exposure, with magnified insets showing lesion progression over time and across the electric field gradient—from the ablation centre (highest field intensity) to the periphery (minimal field intensity).

The interval between exposure to PEFs and the onset of cell death can vary depending on the cell type and pulse parameters.^28,40,41,47,48^ Our previous studies have shown that cardiomyocyte death can continue to develop over a 24 h period following exposure.^40,41^ To determine whether there is a dependence on the applied pulse duration and differences between endothelial and cardiac cells, we tracked the progression of cell death in human cardiomyocytes and endothelial cells after both nsPEF and µsPEF exposure (*Figure 2*), analysing the time-dependent changes in % of dead cells in ROIs.

The increasing percentage of dead cells within the ROI reflects the progression and extent of the electric lesion induced by PEF exposure. In human cardiomyocytes, cell death continued to rise over 24 h following both nsPEF and µsPEF protocols (*Figure* *2A* and *B*). In contrast, in human endothelial cells, cell death plateaued within 6 h after exposure to either nsPEF or µsPEF, indicating a more rapid onset of cytotoxicity in this cell type (*Figure* *2A* and *B*).

For example, after exposure to nsPEF at 8 kV/cm, the percentage of dead cardiomyocytes increased from 26.8% ± 6 at 2 h to 83.5% ± 13 at 24 h (*P* < 0.001). Similarly, following µsPEF, cardiomyocyte death increased progressively across most electric field strengths up to 24 h. The highest fold increase (2.4-fold) was observed at 1.5 kV/cm, where the percentage of dead cardiomyocytes rose from 31.6% ± 7 at 2 h to 76% ± 15 at 24 h (*P* < 0.05). The apparent decline in cell death between 24 and 60 h likely reflects repopulation of the ablated area by viable cells and/or detachment of dead cells. This further decline is illustrated in representative microscopy images provided in Supplementary material online, *Figure S1*.

In endothelial cells, the majority of cell death occurred within the first 2–4 h, with only a minor increase at later time points. Notably, at the highest tested nsPEF (11 kV/cm), cell death reached 63% ± 5 at 2 h and further increased to 95.5% ± 2 by 6 h. At the lowest electric field strength, the percentage of dead endothelial cells decreased over time—from 32% ± 8 at 2 h—likely due to repopulation of the ablated area by viable cells and/or detachment of dead cells.

A similar trend was observed following µsPEF: most endothelial cell death occurred within the first 2–4 h and slightly declined thereafter. For instance, at 2 kV/cm, the percentage of dead cells rose from 74% ± 6 at 2 h to 88% ± 3 at 4 h, followed by a slight decrease to 86% ± 5 at 6 h.

These findings confirm that PEF-induced cell death exhibits distinct, cell-type-specific kinetics, with cardiomyocytes showing delayed responses compared to endothelial cells. Notably, our data suggest that the overall dynamics of cell death were less dependent on the pulse parameter itself.

### *Ex vivo* model—lesion formation dynamics

**Figure 3:**
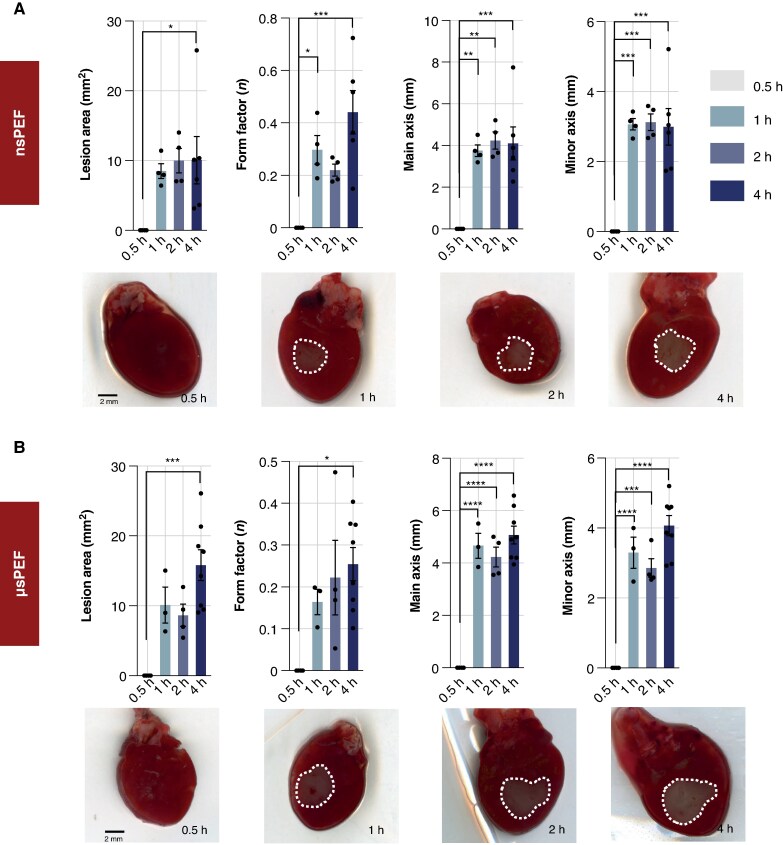
Lesion formation in *ex vivo* murine myocardium. (*A* and *B*) Lesion development in *ex vivo* murine myocardium following epicardial ablation with nsPEF (*A*) and µsPEF (*B*). Ablated regions were visualized using TTC staining at multiple post-exposure time points. White-dashed circles indicate the ablation zones. A time-dependent increase in lesion size was observed for both pulse modalities. Quantitative analysis was conducted using machine learning–based image processing algorithms (see Methods). Data are presented as mean ± SEM from three to eight independent experiments per time point. Key lesion parameters, including area, form factor, and major/minor axis lengths, were evaluated. For µsPEF exposure, lesion area and axis lengths progressively increased, accompanied by a rise in form factor, indicating more defined and stable lesion morphology over time. Similarly, nsPEF treatment produced significant changes in lesion size, shape, and form factor within the first 4 h post-exposure.

In our *ex vivo* studies using murine myocardium, we compared the time-dependent lesion formation following ablation with nsPEF and µsPEF (*Figure* *3A* and *B*). Our objective was to identify the timeframe required to achieve a visibly distinct ablation zone with each protocol.

Nanosecond pulsed electric field ablation led to the formation of visible lesions over time. A detectable lesion area appeared as early as 1 h post-exposure, with significant changes in size and morphology observed up to 4 h (*Figure 3A*). No visible lesion was detected at 0.5 h, whereas by 4 h, the lesion area reached 10.1 ± 3.4 mm² (*P* < 0.05), accompanied by a significant increase in form factor to 0.44 (*P* < 0.001). The major and minor axis lengths also increased significantly, reaching 4.1 ± 0.8 mm and 3.0 ± 0.5 mm, respectively, at 4 h (both *P* < 0.001).

Likewise, exposure to µsPEF resulted in a progressive increase in lesion size over time. The ablation area expanded significantly between 0.5 and 4 h post-exposure, increasing from undetectable at 30 min to 15.8 ± 2.2 mm² at 4 h (*P* < 0.001; *Figure 3B*). This expansion was accompanied by a significant increase in form factor, indicating enhanced uniformity of lesion shape over time (*P* < 0.05). Similarly, both the major and minor axis lengths grew significantly, reaching 5.1 ± 0.3 mm and 4.1 ± 0.3 mm at 4 h, respectively (*P* < 0.05 for both).

Studies using *ex vivo* models have also demonstrated a prolonged cell death process in cardiomyocytes following PEF exposure. At 4 h post-treatment, the lesions remained well-defined, with sharp margins separating viable, TTC-stained tissue from non-viable, unstained areas. At later time points, however, these margins became increasingly diffuse, likely due to early metabolic changes in the non-perfused tissue, which resulted in diminished TTC staining intensity.

### Endothelial cells show higher susceptibility to microsecond pulsed electric fields compared to cardiomyocytes

Following the determination of cell killing time, we compared the susceptibility of human cardiomyocytes and human endothelial cells to nsPEF and µsPEF.

Direct comparison of nsPEF and µsPEF is complicated by the strength–duration relationship of membrane charging, which requires higher field amplitudes for shorter pulses to reach the electroporation threshold.^49^ It is important to note that we did not directly compare nsPEF and µsPEF in terms of killing efficiency at the same electric field strength, as the parameters for the two modalities were necessarily different. Instead, for each modality separately, we applied identical electric field parameters to cardiomyocytes and endothelial cells, allowing us to assess cell type–specific sensitivity to nsPEF and, in a separate analysis, to µsPEF.

**Figure 4:**
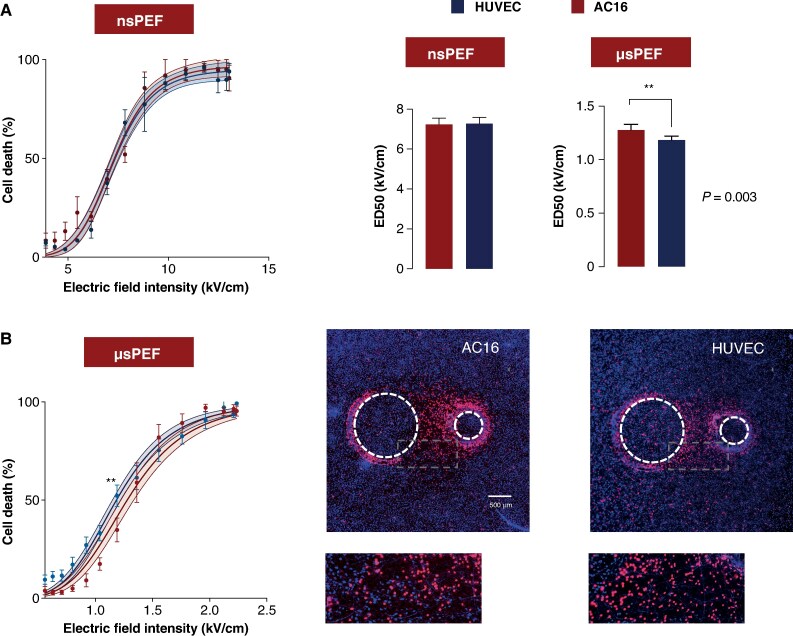
Differential sensitivity of human cardiomyocytes and endothelial cells to µsPEF and nsPEF. (*A* and *B*) Dose–response curves showing the viability of human cardiomyocytes (AC16) and human endothelial cells (HUVEC) following exposure to nsPEF (*A*) and µsPEF (*B*). Data were fitted with sigmoidal curves using the Hill equation; shaded regions represent 95% confidence intervals. Each curve is based on at least five independent experiments. For µsPEF exposure, the ED₅₀ (electric field intensity causing 50% cell death) was significantly lower in HUVECs compared to AC16 cells, indicating greater sensitivity of endothelial cells to µsPEF-induced cytotoxicity. Bar graphs compare the mean ED₅₀ values for AC16 and HUVEC cells after exposure to nsPEF and µsPEF. Error bars indicate 95% confidence intervals. These comparisons further underscore the differential susceptibilities of the two cell types, with endothelial cells showing greater sensitivity to µsPEF. Representative fluorescence microscopy images show peripheral ablation zones in AC16 and HUVEC monolayers after µsPEF exposure. Insets highlight differences in cell viability and lesion extent, illustrating the distinct responses of each cell type to pulsed electric field exposure.

As shown in *Figure 4A*, exposure to nanosecond pulses resulted in similar mortality rates for both cell types across various electric field intensities. The ED_50_ for AC16 was determined to be 7.24 kV/cm (95% CI: 6.9–7.6), while HUVEC exhibited a slightly higher ED_50_ of 7.27 kV/cm (95% CI: 7.0–7.6).

In contrast, when ablated with microsecond pulses, endothelial cells demonstrated significantly greater susceptibility (*Figure 4B*). The ED_50_ for HUVEC was statistically lower than that for AC16 cardiomyocytes, with values of 1.18 kV/cm (95% CI: 1.1–1.2) compared to 1.28 kV/cm (95% CI: 1.2–1.3) for cardiomyocytes (*P* < 0.01).

While these findings highlight variations in susceptibility based on pulse duration, our further research focused on uncovering the underlying mechanisms responsible for the differences in cell killing selectivity between nanosecond and microsecond pulses.

### Interplay between cell death and plasma membrane permeabilization after nanosecond pulsed electric fields vs. microsecond pulsed electric fields

Nanosecond pulsed electric fields and µsPEF generate pores of different sizes in the plasma membrane, with nsPEF producing smaller pores compared to those induced by µsPEF.^43,50–58^

Recent advances in the understanding of electroporation indicate that, regardless of the extent of membrane disruption, cells typically restore membrane continuity within minutes after exposure.^59^ Subsequent cell death arises from metabolic and structural changes that occur during the transient permeabilized state.

**Figure 5:**
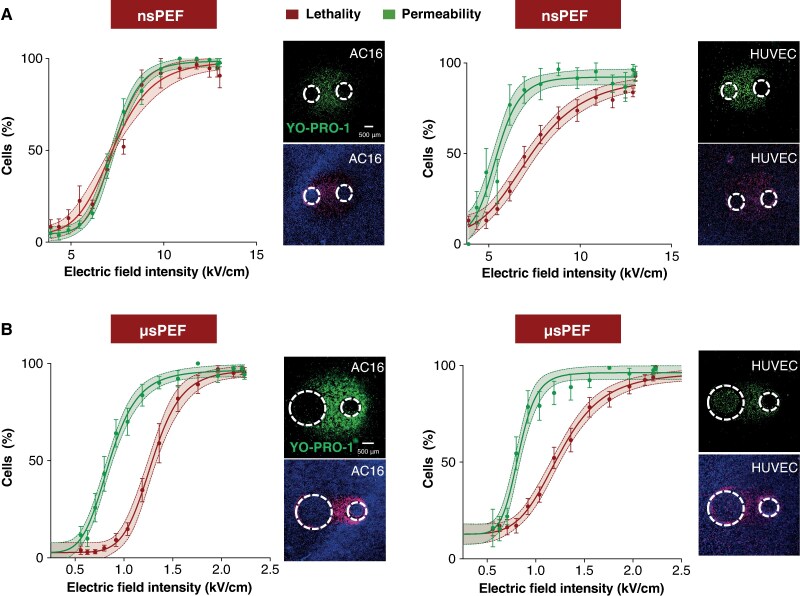
Interplay between plasma membrane permeabilization and ablation efficiency after nsPEF and µsPEF exposure in cardiomyocytes and endothelial cells. (*A* and *B*) The association between plasma membrane permeabilization and cell death in AC16 cardiomyocytes and HUVEC endothelial cells following exposure to nsPEF (*A*) and µsPEF (*B*). Green curves show the extent of membrane permeabilization, quantified by YO-PRO-1 uptake as a function of calculated electric field intensity across ROIs. Red curves indicate cell death, measured by PI uptake at the peak time point determined from the cell death time-course analysis (see *Figure 2*). Data were fitted using sigmoidal curves generated with the Hill equation (solid lines), and shaded regions represent 95% confidence intervals. Data are presented as mean ± SEM, *n* = 3–6 independent experiments per fit. Non-overlapping confidence intervals indicate statistically significant differences between permeabilization and lethality curves (*P* < 0.05). Representative images show the ablation zones in cell monolayers, highlighting cell death (red/PI signal) and permeabilization (green/YO-PRO-1 signal) after nsPEF and µsPEF exposure. White circles mark electrode footprints. These images visualize the spatial distribution of permeabilized vs. lethally ablated cells across the electric field gradient.

To investigate the relationship between cell death and plasma membrane permeabilization, we used YO-PRO-1 dye staining to quantify membrane permeability. In human cardiomyocytes exposed to nanosecond electroporation (*Figure 5A*), plasma membrane permeabilization led directly to the death of the permeabilized cells. In AC16 cells, the ED₅₀ for permeabilization was 7.29 kV/cm (95% CI: 7.11–7.48), closely matching the ED₅₀ for lethality, which was 7.43 kV/cm (95% CI: 6.98–7.88). A comparable pattern was observed in HUVEC cells exposed to nanosecond pulses (*Figure 5A*), where the ED₅₀ for membrane permeabilization was 5.43 kV/cm (95% CI: 5.10–5.75), while for cell death, it was higher, at 7.28 kV/cm (95% CI: 6.62–7.84).

During electroporation with longer microsecond pulses, membrane permeability in both cardiomyocytes and endothelial cells exceeded cell lethality (*Figure 5B*). Specifically, in AC16 cardiomyocytes the ED₅₀ for membrane permeabilization was 0.85 kV/cm (95% CI: 0.82–0.89), while the ED₅₀ for cell death was 1.28 kV/cm (95% CI: 1.24–1.34). Similarly, in HUVEC endothelial cells treated with µsPEF, the ED₅₀ for permeabilization was 0.82 kV/cm (95% CI: 0.79–0.86) and for lethality 1.25 kV/cm (95% CI: 1.18–1.31).

Our findings demonstrate that pulse duration strongly influences how membrane permeabilization translates into cell death, potentially explaining the divergent responses of cardiomyocytes and endothelial cells to nsPEF and µsPEF. Notably, while nsPEF-induced permeabilization in cardiomyocytes consistently led to cell death, µsPEF exposure did not elicit the same outcome. This key observation led us to further investigate the downstream molecular pathways activated in cardiomyocytes following nsPEF and µsPEF treatment.

### Raman spectroscopy shows the molecular changes corresponding to activation of regulated cell death after exposure to nanosecond pulsed electric fields

**Figure 6:**
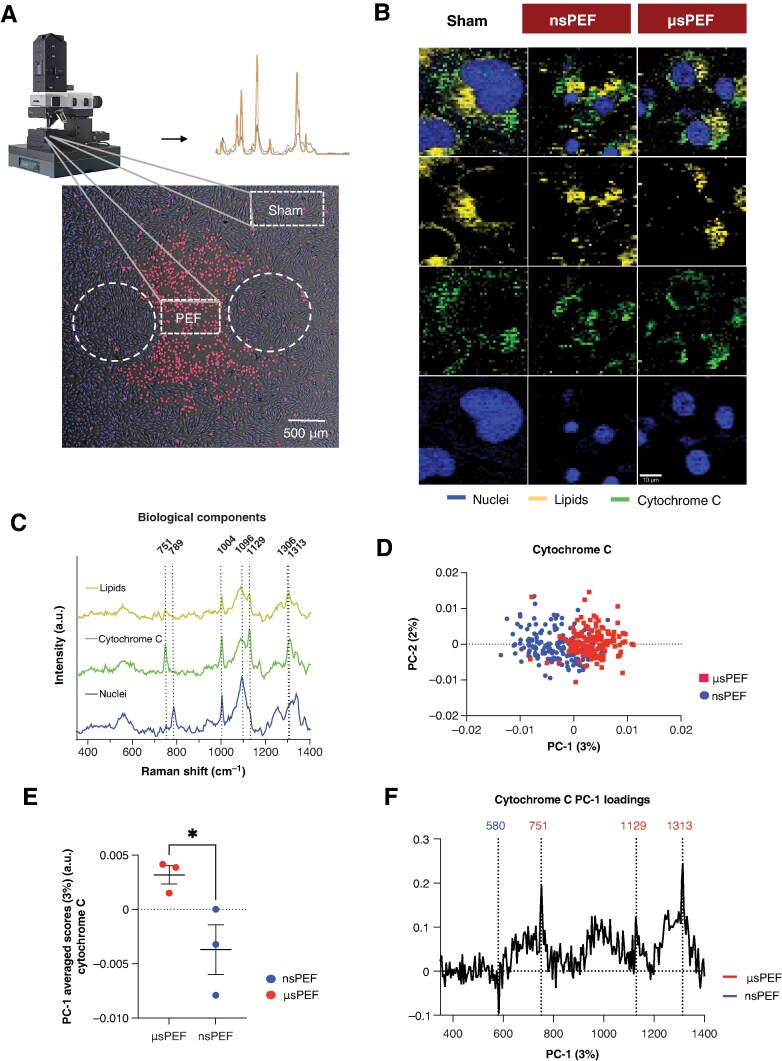
Multivariate spectral analysis reveals molecular differences between nsPEF- and µsPEF-induced ablation in human cardiomyocytes. (*A*) Overview of the experimental workflow and region selection for Raman spectroscopy. Human cardiomyocytes were cultured in removable eight-well chambers pre-coated with human fibronectin, electroporated with either nsPEF or µsPEF, and incubated for 4 h. Raman imaging was performed using a confocal microspectrometer. A representative brightfield image shows a cardiomyocyte monolayer with a clearly marked ablation region (PEF) and a non-ablated control region (sham) on the periphery. These regions were selected for Raman acquisition based on proximity to the electrode footprint (white-dashed lines) and morphological changes consistent with electroporation. (*B*) TCA of the spectral maps of healthy and ablated by nsPEF and µsPEF cells (blue: nuclei, yellow: lipids, green: cytochrome c), showing spatial patterns and relative expression levels. Scale bar = 10 µm. (*C*) Reference Raman spectra of cellular components (nuclei, lipids, cytochrome c). (*D*) PCA of cytochrome c spectra demonstrates distinct separation between nsPEF- and µsPEF-treated groups in the PC1 vs. PC2 scores plot. (*E*) Significant separation along the PC1 axis between nsPEF and µsPEF groups (*P* = 0.0484), indicating distinct molecular signatures. (*F*) PC1 loadings plot highlight the spectral features that contribute most to group separation; positive peaks reflect features enriched in the nsPEF group, while negative peaks correspond to µsPEF-associated signatures. Statistical analysis of the average PC1 scores revealed a significant difference between the nsPEF and µsPEF groups (*n* = 3 per group, unpaired *t*-test, mean ± SD). Notably, spectral differences were observed at multiple cytochrome c peaks, including increased signals at 751 cm⁻¹ and 1129 cm⁻¹ in µsPEF-treated cells (associated with the reduced Fe²⁺ form) and a prominent 580 cm⁻¹ peak in nsPEF-treated cells (associated with the oxygenated form). These findings suggest that µsPEF exposure favours accumulation of the reduced form of cytochrome c, potentially suppressing apoptosis and promoting acute cell death, whereas nsPEF induces higher levels of the oxidized form, facilitating apoptotic processes.

To specifically investigate the mechanisms of cell death, we selected cells within the ROI between electrodes corresponding to an electric field intensity between 12.5–13 kV/cm for nsPEF and 2.1–2.2 kV/cm for µsPEF. To capture the progression of cell death and potential differences between microsecond and nanosecond pulse exposures, cells were fixed 4 h post-exposure for subsequent Raman analysis. Images were acquired in the ablated region as well as in the periphery (*Figure 6A*).

Analysis of hyperspectral maps enabled marker-independent localization of subcellular structures and visualization of cell morphology. Three cellular components were discriminated via TCA by specific spectral signatures: nuclei (blue), lipids (yellow), and cytochrome c (green) (*Figure* *6B* and *C*). The resulting false colour-coded intensity distribution heat maps represent the distribution patterns and expression levels of components. Nuclei spectrum was identified by peaks at 789 cm^−1^ and 1096 cm^−1^ which are related to phosphodiester (PO_2_^−^) bands in DNA (see Supplementary material online, *Table S1*). Spectrum of lipids was verified by characteristic peaks at 1089 cm^−1^ and 1127 cm^−1^ which correspond to C-C stretching bands as well as 1306 cm^−1^ CH_2_ twisting bands. Cytochrome c, which is a small heme protein located in the mitochondrial intermembrane space, was assigned to main bands 751 cm^−1^ and 1129 cm^−1^ which indicate pyrrole breathing band and symmetrical pyrrole half-ring band.

To further assess submolecular changes, individual spectra were extracted for every component and further analysed by PCA to evaluate spectral alterations between the PEF treatments. Principal component analysis of nuclei and lipid spectra did not reveal significant differences between the two groups (see Supplementary material online, *Figure S2*). The PC-1 vs. PC-2 scores plot for cytochrome c demonstrated a cluster formation between cytochrome c signatures of cardiomyocytes ablated with nsPEF and µsPEF (*Figure 6D*). The data from the nsPEF group show a significant shift towards the positive PC1 range compared to the µsPEF group (*Figure 6E*). In the corresponding loadings plot, positive peaks reflect dominant spectral features in data with positive PC1 scores, while negative bands highlight spectral signatures associated with clustering in the negative score range (*Figure 6F*). The PC1 loadings plot highlights molecular features responsible for the spectral differences, indicated by peak shifts at specific wavenumbers.

Spectral differences were observed at multiple peak positions. The biological assignments of the Raman spectra are provided in Supplementary material online, *Table S1*. For cytochrome c, significantly increased peaks at 751, 1129, and 1313 cm^−1^, representing pyrrole ring bands, were observed in cells ablated with µsPEF. In contrary, the 580 cm^−1^ peak showed a stronger intensity in cells ablated with nsPEF and is responsible for Fe–O₂ stretching mode. Cytochrome c exists in two redox states, defined by the oxidation state of the central iron atom in its heme group. Whereas the 751 and 1129 cm^−1^ bands are characteristic features of its reduced form (Fe²⁺),^60^ the 580 cm^−1^ band corresponds to the oxygenated form of cytochrome c.^61^ Averaged signal intensity of 751 cm^−1^ peak was significantly increased for the µsPEF group compared to the nsPEF and sham group (see Supplementary material online, *Figure S3*).

## Discussion

The use of PEFs to selectively target the myocardial cellular compartment presents a promising strategy for treating cardiac arrhythmias. Currently, clinical PFA predominantly uses mono- and bipolar microsecond pulses. As a novel modality of cardiac ablation, nanosecond PFA is currently undergoing initial clinical trials (NCT06355063).

In this study, we demonstrate a significant difference in the relationship between plasma membrane permeabilization and cell death induced by nano- vs. microsecond pulses. In cardiomyocytes, membrane permeabilization caused by nsPEF consistently led to cell death. In contrast, cells exposed to microsecond pulses can remain viable despite evident membrane permeabilization.

It is known that nsPEF requires higher voltages to achieve electroporation than µsPEF due to the strength–duration relationship of membrane charging.^49^ Short pulses end before the membrane reaches its full depolarization potential, necessitating stronger fields for electroporation. A key advantage is that nsPEFs, being much shorter than the membrane charging time constant, are inefficient at activating voltage-gated ion channels, thereby minimizing off-target neuromuscular and vascular stimulation while still inducing nanoporation.^8,49,59,62,63^ To compare µsPEF and nsPEF meaningfully, we analysed biological effects across the wide range of field strengths for each modality separately, deriving ED₅₀ values for permeabilization and killing. This approach allowed assessment of cell response without conflating differences in absolute field magnitude, providing clinically relevant insights as both modalities enter wider use.

Most clinically available PFA systems deliver monophasic or biphasic microsecond-range pulses (≈500–2000 V) optimized to create deep myocardial lesions with specific electrode geometries.^64,65^ However, key waveform and pulse train parameters (e.g. repetition rate, pulses per burst) are typically undisclosed by manufacturers and are not directly transferable to single-cell or monolayer experiments. Likewise, the company developing an nsPEF catheter for cardiac ablation has not released its pulse specifications. Based on preclinical reports, the pulse width is approximately 200–400 ns—substantially shorter than other available generators.^66,67^

To enable a fair comparison of nsPEF and µsPEF ablation after setting a fixed pulse duration, we adjusted the pulse count, amplitude (voltage), and repetition rate to produce measurable, size-comparable lesions in cell monolayers, equalize total pulse train exposure time across conditions, and minimize Joule heating by using low repetition rates with sufficient inter-pulse intervals for heat dissipation. The work of Pakhomov *et al*.^68^ demonstrated that pulse frequency has no substantial biological impact when it is below the threshold for summation of transmembrane voltage—that is, when the interval between pulses exceeds the membrane relaxation time (approximately 10 µs, corresponding to ∼0.1 MHz). In our experiments, the pulse repetition rates (1 Hz for µsPEF and 10 Hz for nsPEF) were selected to yield an identical total pulse train duration of 20 s. Higher frequencies were avoided to minimize the risk of cumulative heating and thermal injury, ensuring that the observed effects were attributable to electroporation rather than temperature-induced cell damage. Although clinical devices may differ in pulse width and repetition rate, our protocols preserved the >100-fold difference in pulse duration that distinguishes nsPEF from µsPEF technologies.

The findings of our study may have clinical implications regarding the stunning phenomenon during PFA.^69^ Stunning occurs when a group of injured but not fully destroyed cells temporarily loses electrical conduction, which can complicate the evaluation of ablation effectiveness. It is known that cells experiencing mild electroporation can survive regaining normal physiological function. At electroporated state, the cardiomyocyte will eventually stop developing an action potential as shown in the research of Pakhomov group where repetitive stimulation of cardiomyocytes with short electric pulses has caused the electroporation of membrane and resistance to further excitation.^39,70^ Clinically, electroporation causes a reduction in the amplitude of action potentials and a slower rate of rise in their upstroke (dV/dt).^71^ Once these temporarily stunned cells recover their membrane integrity, they can survive, retain their conductive properties, and contribute to the recurrence of arrhythmias.

Our research showed that the amount of reversibly electroporated cardiomyocytes after application of nsPEF is minimalized whereas after µsPEF the substantial proportion of cells survive the transient permeabilization potential regaining the ability for further excitation thus creating the substrate for arrhythmia recurrence.

Moreover, our data indicate that µsPEFs cause more pronounced endothelial injury than nsPEF. Endothelial injury diminishes antithrombotic signalling (e.g. nitric oxide, prostacyclin), increases oxidative stress and ROS, and initiates tissue fibrosis that may promote electrical decoupling.^32,33^ Together, these inflammatory processes drive post-ablation tissue remodelling, a major contributor to atrial fibrillation recurrence.^29–31^

Our demonstration of greater endothelial damage after µsPEF exposure supports the hypothesis that µsPEF amplify local inflammation and, in turn, increase the risk of AF recurrence. Although a mechanistic dissection of the downstream consequences of PEF-induced endothelial injury lies beyond the scope of this study, it remains an important direction for future research.

The mechanisms of cell death following membrane permeabilization have been investigated across various cell lines and electroporation protocols. Several studies have confirmed the occurrence of apoptosis; however, factors such as the composition of the extracellular medium and the concentration of Ca^2+^ significantly influence the predominant mode of cell death observed.^47,72,73^

The key mechanisms involved in cell injury include (i) damage to DNA and proteins,^28^ (ii) elevated levels of ROS,^74^ (iii) increased intracellular calcium,^47,72,73,75^ (iv) mitochondrial impairment,^73,74^ and (v) depletion of ATP.^76,77^ Often, a single harmful stimulus can trigger several of these pathways simultaneously.^78^ This complexity highlights the challenge of pinpointing a single mode of intervention responsible for cellular damage during electroporation.

Raman spectroscopy is a non-invasive, marker-independent technique well-suited for detecting cytochrome c in biological samples, including cells and tissues. Its sensitivity to heme group vibrations allows differentiation between reduced and oxidized forms of cytochrome c, notably through distinct Raman peak near 751 cm⁻¹.^79^ This enables marker-independent monitoring of cytochrome c dynamics and redox state during processes such as apoptosis,^80^ necrosis,^81^ cancer progression,^82–84^and tissue metabolism.^85,86^

In our study, cytochrome c was localized and investigated with Raman imaging. Further analysis of the spectral information revealed significant molecular changes indicating shifts between the reduced and oxidized status of the molecule. In response to cellular stress or damage, cytochrome c is released from the mitochondria into the cytosol, where it triggers apoptosome formation, leading to the activation of caspase-9 and caspase-3. Notably, only the oxidized form of cytochrome c can initiate this pathway, whereas the reduced form fails to activate it, effectively inhibiting apoptosis.^87^ The redox state of cytochrome c is shown to be unchanged during its release from the mitochondria throughout the process of apoptosis.^80^

Moreover, cytochrome c also exhibits peroxidase activity during apoptosis. When cytochrome c binds to cardiolipin on the inner mitochondrial membrane, its conformation changes, exposing the heme group and enhancing its ability to catalyse the reduction of hydrogen peroxide (H₂O₂). This leads to the oxidation of cardiolipin, a critical step in mitochondrial membrane permeabilization and the subsequent release of cytochrome c into the cytosol that induces apoptosis.^88,89^

Our findings, supported by the literature, suggest that exposure of cells to µsPEF increases the levels of the reduced form of cytochrome c, which inhibits apoptosis and might promote a more acute and uncontrolled form of cell death. While in cells ablated by nsPEF an opposite tendency is observed, higher levels of oxidized cytochrome c are present, facilitating the activation of apoptosis.

This observation is supportive but not definitive, as Raman spectroscopy alone cannot conclusively determine cell death type, and confirmation with complementary biochemical and imaging approaches will be required in future.

In our studies, we used experimentally tractable, widely adopted models—AC16 cardiomyocyte and human umbilical vein endothelial cells HUVEC. Both cell lines were immortalized, and both models were maintained under proliferative culture conditions. Consequently, they not fully recapitulate the phenotype or microenvironment of adult human cardiomyocytes or endothelial cells. These cells were well suited to our monolayer model as their strong adhesion and stable, confluent growth enabled controlled analyses. However, the absence of native 3D architecture, vascular-wall context, and tissue-level conductivity limits direct extrapolation of single-cell ED₅₀ values to intact myocardium or vasculature.

In recent years, significant progress in cardiac mapping has improved the precision and completeness of ablation procedures.^90^ Parallel advances in ablation technologies have focused on achieving deeper and more controlled tissue penetration. In the context of evolving clinical strategies for durable and selective cardiac ablation, nsPEF can produce more homogeneous lesions because their interaction with cell membranes differs fundamentally from that of longer pulses. Micro- to millisecond pulses drive the movement of free electrolyte charges in the applied electromagnetic field. As cell membranes act as barriers to ion flow, these charges accumulate on the membrane surfaces, leading to capacitive charging.^67,91^ In cardiac tissue, intercellular electrical coupling restricts free ion movement and limits uniform charging, so electroporation occurs predominantly near the cathode and at sites of abrupt conductivity changes—such as fibre junctions, fat inclusions, or scar tissue—where local potential drops form ‘virtual cathodes’.^67,92^ In contrast, nsPEFs are too brief for substantial capacitive charging, and displacement currents dominate over conduction currents. The rapid field change prevents significant ionic movement; instead, bound charges in the membrane polarize without large-scale ion transport. This minimizes membrane charging via ionic conduction, allowing the field to penetrate more uniformly and deeply, thereby generating more homogeneous lesions.^67,93,94^

The observed differences in cell sensitivity and cell death mechanisms underscore the potential advantages of nsPEF for cardiac ablation. A deeper understanding of how pulse duration influences both cardiac selectivity and efficacy can improve the protocols and therapeutic outcomes.

## Supplementary Material

euaf217_Supplementary_Data

## Data Availability

The data underlying this article will be shared on reasonable request to the corresponding author.
