# Supplementary material for: Impact of pulse duration on cardiac electroporation: nanosecond pulses enhance cardiomyocyte selectivity and promote a Raman-detected shift towards apoptotic cell death
Source: Europace. 2025 Sep 13;27(9):euaf217. doi: 10.1093/europace/euaf217 (PMC12481159; doi:10.1093/europace/euaf217)
Supplement: euaf217_Supplementary_Data [file euaf217_supplementary_data.zip › Supplementary Materials.docx]

**Supplementary information to**

**Impact of Pulse Duration on Cardiac Electroporation: Nanosecond Pulses Enhance Cardiomyocyte Selectivity and Promote a Raman-Detected Shift Toward Apoptotic Cell Death**

Authors: Pamela W. Sowa^1^, MD, Aleksandra Mariyanats^2^, MS, Aleksander Kiełbik^3^, MD, PhD, Anne-Katrin Rohlfing^1^ PhD, Vitalij Novickij^4,5^, PhD, Ferdinand Kollotzek^1,6^, MS, Manuel Sigle^1^, MD, Julia Marzi^2,7^, PhD, Katja Schenke-Layland,^2,7^ PhD, Oliver Borst^1,6^, MD, Meinrad P. Gawaz^1^, MD

^1^University Hospital Tübingen, Department of Cardiology and Angiology, Tübingen, Germany

^2^Institute of Biomedical Engineering, Department for Medical Technologies and Regenerative Medicine, Eberhard Karls University Tübingen, Tübingen, Germany

^3^University Hospital Tübingen, Department of Urology, Tübingen, Germany

^4^Vilnius Gediminas Technical University, Institute of High Magnetic Fields, Vilnius, Lithuania

^5^State Research Institute Centre for Innovative Medicine, Department of Immunology and Bioelectrochemistry, Vilnius, Lithuania

^6^DFG Heisenberg Group Cardiovascular Thrombo-Inflammation and Translational Thrombocardiology, University of Tübingen, Tübingen, Germany

^7^NMI Natural and Medical Sciences Institute at the University of Tübingen, Reutlingen, Germany

Correspondence to: Pamela W. Sowa, MD, University Hospital Tübingen, Department of Cardiology and Angiology, Otfried-Müller-Straße 10 72076 Tübingen, Germany, Email pamela.sowa@med.uni-tuebingen.de

Table of content

| **Content** | **Description** | **Page** |
| --- | --- | --- |
| Supplementary  Table **S1** | **Raman shifts and their molecular assignments** | 4-8 |
| Supplementary  Figure **S1** | **Representative fluorescence microscopy images of human cardiomyocyte monolayers following nsPEF and µsPEF exposure at 30, 48, and 60 hours post-treatment.** | 9 |
| Supplementary Figure **S2** | **Raman spectral characterization and principal component analysis of nuclear and lipid components following nsPEF and µsPEF exposure** | 10 |
| Supplementary Figure **S3** | **Increased intensity of 751 cm^-1^ peak in µsPEF group reveals enhanced content of reduced form of cytochrome c** | 11 |

**Table S1. Raman shifts and their molecular assignments**

| Raman Shift [cm^−1^] | Molecular Assignment | Structure | References |
| --- | --- | --- | --- |
| 580 | Fe–O₂ stretching mode | Cytochrome c | Zhang, Mohan, et al. "Resonance Raman Studies on Heme Ligand Stretching Modes in Methionine80-Depleted Cytochrome c: Fe–His, Fe–O2, and O–O Stretching Modes." The Journal of Physical Chemistry B 127.11 (2023): 2441-2449. |
| 751 | Heme breathing | Cytochrome c | [Strekas, Thomas C., and Thomas G. Spiro. “Cytochrome c: resonance Raman spectra.” Biochimica et Biophysica Acta (BBA)-Protein Structure 278.1 (1972): 188-192.](https://doi.org/10.1016/0005-2795(72)90121-3)  Hu, Songzhou, et al. "Complete assignment of cytochrome c resonance Raman spectra via enzymic reconstitution with isotopically labeled hemes." Journal of the American Chemical Society 115.26 (1993): 12446-12458.  Adar, Fran, and Maria Erecińska. "Resonance Raman spectra of the b-and c-type cytochromes of succinate-cytochrome c reductase." Archives of Biochemistry and Biophysics 165.2 (1974): 570-580. |
| 789 | Phosphodiester bands | DNA | Prescott, B., W. Steinmetz, and G. J. Thomas Jr. "Characterization of DNA structures by laser Raman spectroscopy." Biopolymers: Original Research on Biomolecules 23.2 (1984): 235-256.  Benevides, J. M., et al. "Crystal and solution structures of the B-DNA dodecamer d (CGCAAATTTGCG) probed by Raman spectroscopy: heterogeneity in the crystal structure does not persist in the solution structure." Biochemistry 27.3 (1988): 931-938. |
| 1004 | Phenylalanine | Proteins | Hernández, Belén, et al. "Characteristic Raman lines of phenylalanine analyzed by a multiconformational approach." Journal of Raman Spectroscopy 44.6 (2013): 827-833. |
| 1089 | C - C stretching | Lipids | Czamara, Krzysztof, et al. "Raman spectroscopy of lipids: a review." Journal of Raman spectroscopy 46.1 (2015): 4-20. |
| 1095 | Backbone PO2- | DNA | Prescott, B., W. Steinmetz, and G. J. Thomas Jr. "Characterization of DNA structures by laser Raman spectroscopy." Biopolymers: Original Research on Biomolecules 23.2 (1984): 235-256.  Benevides, J. M., et al. "Crystal and solution structures of the B-DNA dodecamer d (CGCAAATTTGCG) probed by Raman spectroscopy: heterogeneity in the crystal structure does not persist in the solution structure." Biochemistry 27.3 (1988): 931-938. |
| 1127 | C-C stretching | Lipids | Czamara, Krzysztof, et al. "Raman spectroscopy of lipids: a review." Journal of Raman spectroscopy 46.1 (2015): 4-20. |
| 1129 | Symmetrical pyrrole half-ring band | Cytochrome c | [Strekas, Thomas C., and Thomas G. Spiro. “Cytochrome c: resonance Raman spectra.” Biochimica et Biophysica Acta (BBA)-Protein Structure 278.1 (1972): 188-192.](https://doi.org/10.1016/0005-2795(72)90121-3)  Hu, Songzhou, et al. "Complete assignment of cytochrome c resonance Raman spectra via enzymic reconstitution with isotopically labeled hemes." Journal of the American Chemical Society 115.26 (1993): 12446-12458.  Adar, Fran, and Maria Erecińska. "Resonance Raman spectra of the b-and c-type cytochromes of succinate-cytochrome c reductase." Archives of Biochemistry and Biophysics 165.2 (1974): 570-580. |
| 1169 | Symmetrical pyrrole half-ring band | Cytochrome c | [Strekas, Thomas C., and Thomas G. Spiro. “Cytochrome c: resonance Raman spectra.” Biochimica et Biophysica Acta (BBA)-Protein Structure 278.1 (1972): 188-192.](https://doi.org/10.1016/0005-2795(72)90121-3) |
| 1246 | Amide III | Proteins | Lippert, J. L., D. Tyminski, and P. J. Desmeules. "Determination of the secondary structure of proteins by laser Raman spectroscopy." Journal of the American Chemical Society 98.22 (1976): 7075-7080. |
| 1256 | Ring breathing of adenine, cytosine | DNA | Prescott, B., W. Steinmetz, and G. J. Thomas Jr. "Characterization of DNA structures by laser Raman spectroscopy." Biopolymers: Original Research on Biomolecules 23.2 (1984): 235-256.  Benevides, J. M., et al. "Crystal and solution structures of the B-DNA dodecamer d (CGCAAATTTGCG) probed by Raman spectroscopy: heterogeneity in the crystal structure does not persist in the solution structure." Biochemistry 27.3 (1988): 931-938. |
| 1306 | CH2 twisting | Lipids | Czamara, Krzysztof, et al. "Raman spectroscopy of lipids: a review." Journal of Raman spectroscopy 46.1 (2015): 4-20. |
| 1313 | Pyrrole rings vibrations | Cytochrome c | [Strekas, Thomas C., and Thomas G. Spiro. “Cytochrome c: resonance Raman spectra.” Biochimica et Biophysica Acta (BBA)-Protein Structure 278.1 (1972): 188-192.](https://doi.org/10.1016/0005-2795(72)90121-3)  Hu, Songzhou, et al. "Complete assignment of cytochrome c resonance Raman spectra via enzymic reconstitution with isotopically labeled hemes." Journal of the American Chemical Society 115.26 (1993): 12446-12458.  Adar, Fran, and Maria Erecińska. "Resonance Raman spectra of the b-and c-type cytochromes of succinate-cytochrome c reductase." Archives of Biochemistry and Biophysics 165.2 (1974): 570-580. |
| 1340 | Ring breathing of adenine | DNA | Prescott, B., W. Steinmetz, and G. J. Thomas Jr. "Characterization of DNA structures by laser Raman spectroscopy." Biopolymers: Original Research on Biomolecules 23.2 (1984): 235-256.  Benevides, J. M., et al. "Crystal and solution structures of the B-DNA dodecamer d (CGCAAATTTGCG) probed by Raman spectroscopy: heterogeneity in the crystal structure does not persist in the solution structure." Biochemistry 27.3 (1988): 931-938. |
| 1374 | Ring breathing in thymine, adenine, guanine | DNA | Prescott, B., W. Steinmetz, and G. J. Thomas Jr. "Characterization of DNA structures by laser Raman spectroscopy." Biopolymers: Original Research on Biomolecules 23.2 (1984): 235-256.  Benevides, J. M., et al. "Crystal and solution structures of the B-DNA dodecamer d (CGCAAATTTGCG) probed by Raman spectroscopy: heterogeneity in the crystal structure does not persist in the solution structure." Biochemistry 27.3 (1988): 931-938. |

**Supplementary Figure S1.** Representative fluorescence microscopy images of human cardiomyocyte monolayers following nsPEF and µsPEF exposure at 30-, 48-, and 60-hours post-treatment.
(A–B) Time-dependent cell death in cardiomyocytes exposed to nanosecond pulsed electric fields (nsPEF: 200 pulses, 10 Hz, 300 ns, 1.4 kV; Panel A) or microsecond pulsed electric fields (µsPEF: 20 pulses, 1 Hz, 100 µs, 300 V; Panel B). As shown in Figure 2, cardiomyocyte death increased markedly within the first 24 h for both pulse types, with an apparent decline between 24 and 60 h, likely reflecting repopulation of the ablated area by viable cells and/or detachment of dead cells.

**Figure S2. Raman spectral characterization and principal component analysis of nuclear and lipid components following nsPEF and µsPEF exposure**
(A–B) PC loading plots highlight the spectral features that most strongly contribute to group differentiation. Positive peaks indicate features enriched in nsPEF-treated samples, while negative peaks correspond to features associated with µsPEF exposure.
(C–D) PCA scores plots (PC2 vs. PC3 and PC3 vs. PC4) for nuclear and lipid spectra show no distinct clustering, indicating no significant separation between nsPEF and µsPEF groups.
(E–F) This is consistent with the absence of significant submolecular changes in nuclear or lipid composition between the two treatment modalities (n = 3 per group, unpaired *t*-test, mean ± SD).

**Supplementary Figure S3. Increased intensity of 751 cm^-1^ peak in µsPEF group reveals enhanced content of reduced form of cytochrome c** (A) The mean Raman spectra of cytochrome c demonstrate increased intensity of 751 cm^-1^ peak in µsPEF group. (B) Statistical analysis (one-way ANOVA) showed that 751 cm^-1^ peak intensity is significantly higher in µsPEF group in comparison with nsPEF and sham groups
