# Supplementary figures and images for: Impact of pulse duration on cardiac electroporation: nanosecond pulses enhance cardiomyocyte selectivity and promote a Raman-detected shift towards apoptotic cell death
Source: Europace. 2025 Sep 13;27(9):euaf217. doi: 10.1093/europace/euaf217 (PMC12481159; doi:10.1093/europace/euaf217)

A

B

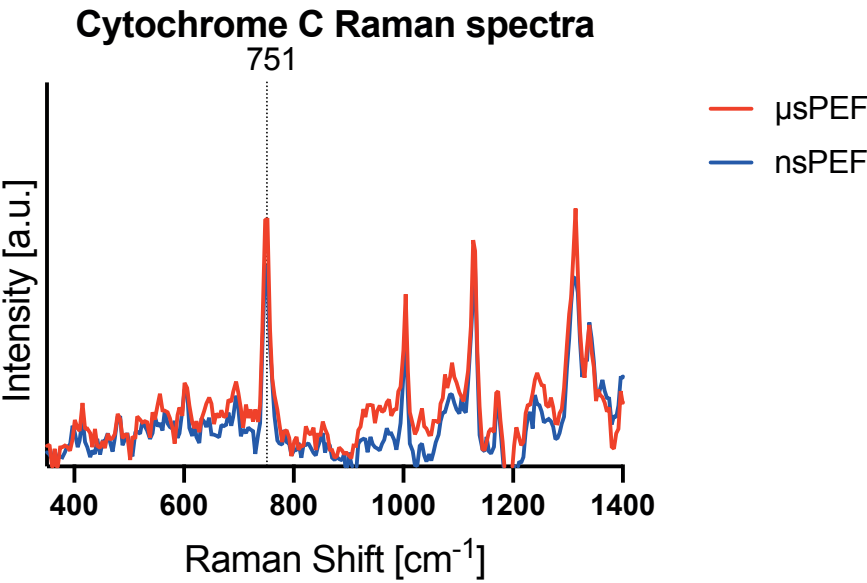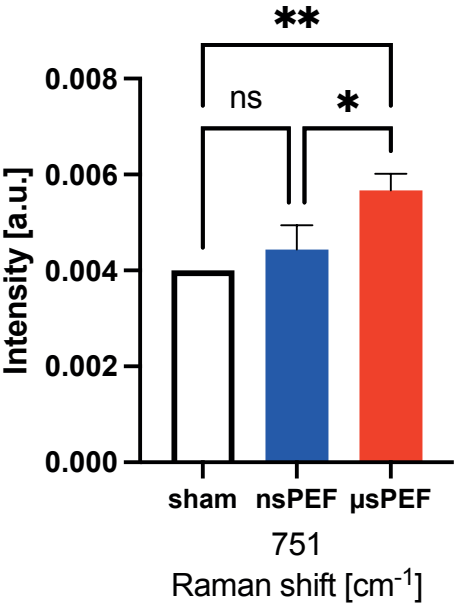

Supplement: euaf217_Supplementary_Data [file euaf217_supplementary_data.zip › Suplemmentary Fig. S3-2.pdf]

**A**

**nsPEF**

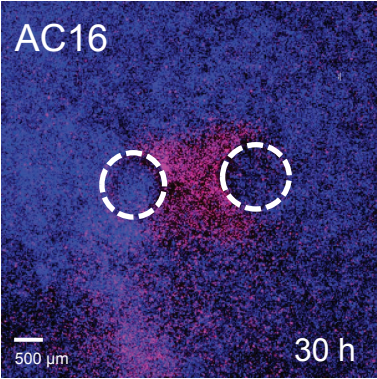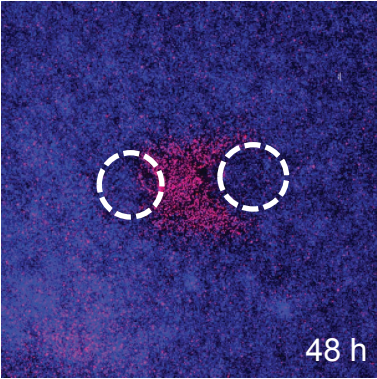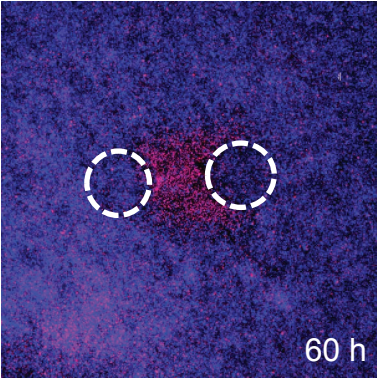

**B**

**$\mu$ sPEF**

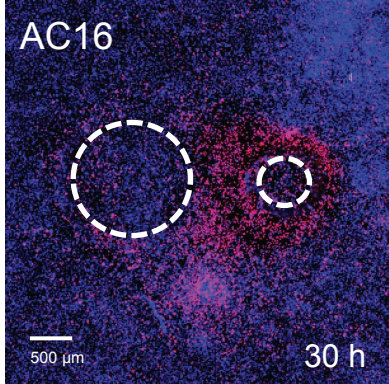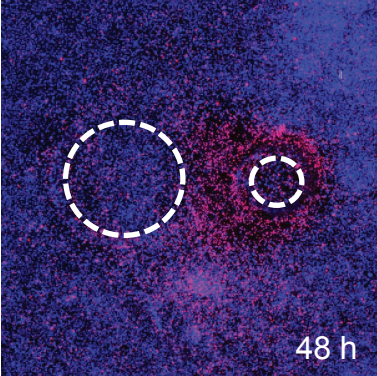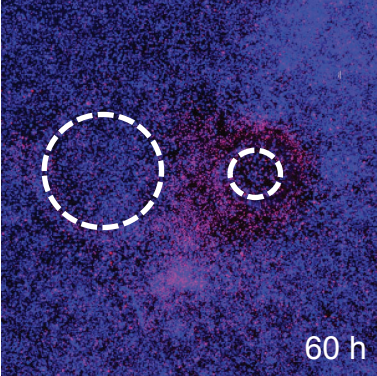

Supplement: euaf217_Supplementary_Data [file euaf217_supplementary_data.zip › Supplementary Fig. S1-2.pdf]

**Lipids PC-2 loadings**

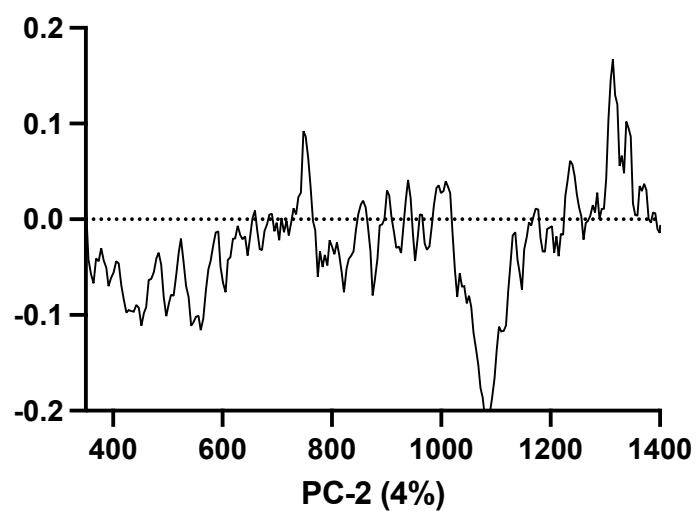

**Nuclei PC-3 loadings**

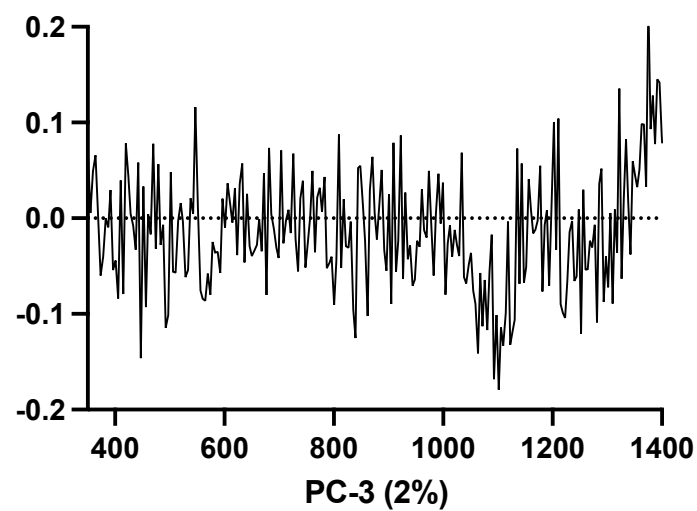

**Lipids**

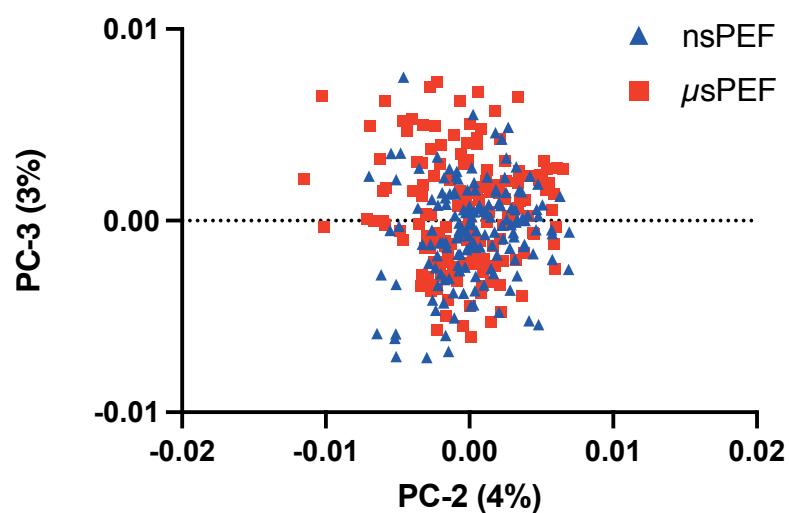

**Nuclei**

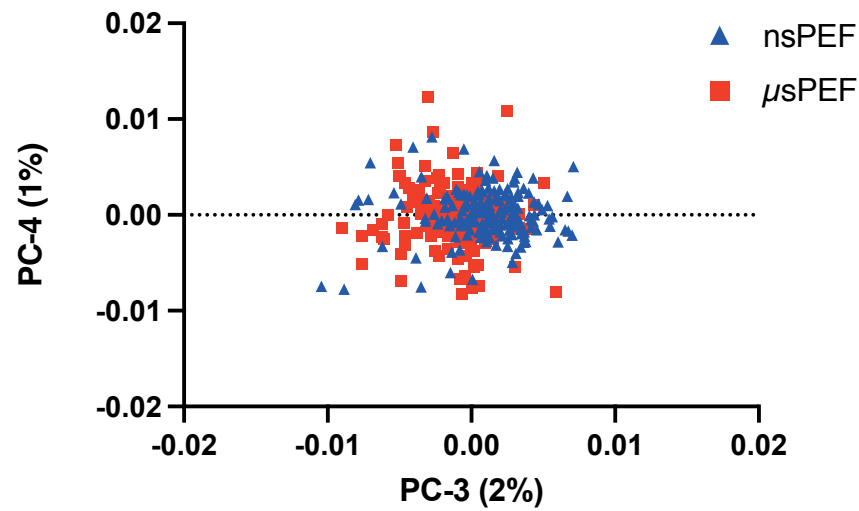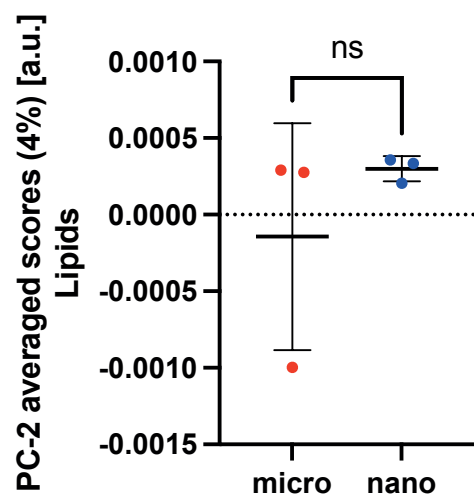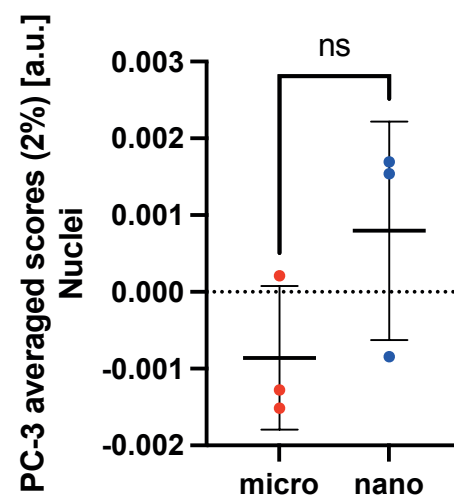

Supplement: euaf217_Supplementary_Data [file euaf217_supplementary_data.zip › Supplementary Fig. S2-2.pdf]
